# Tracking the neurophysiological effects of proteinopathy into the pre-clinical stage of Alzheimer’s disease

**DOI:** 10.1093/braincomms/fcaf069

**Published:** 2025-02-11

**Authors:** Alex I Wiesman, Santiago I Flores-Alonso

**Affiliations:** Department of Biomedical Physiology and Kinesiology, Simon Fraser University, Burnaby, Canada, BC V3L 1B3; Department of Biomedical Physiology and Kinesiology, Simon Fraser University, Burnaby, Canada, BC V3L 1B3

## Abstract

This scientific commentary refers to ‘Amyloid-β deposition predicts oscillatory slowing of magnetoencephalography signals and a reduction of functional connectivity over time in cognitively unimpaired adults’, by Scheijbeler *et al*. (https://doi.org/10.1093/braincomms/fcaf018).

**This scientific commentary refers to ‘Amyloid-β deposition predicts oscillatory slowing of magnetoencephalography signals and a reduction of functional connectivity over time in cognitively unimpaired adults’, by Scheijbeler *et al*. (https://doi.org/10.1093/braincomms/fcaf018).**

Alzheimer’s disease is now viewed as a clinical continuum,^1^ with early pathophysiological processes preceding cognitive symptoms by several years. Recent multimodal neuroimaging research has investigated the neurophysiological reactivity of neuronal populations to these pathological processes, with the hope of deriving a sensitive, non-invasive and repeatable prognostic marker for early-stage Alzheimer’s disease.

In the clinical stages of mild cognitive impairment (MCI) and Alzheimer’s disease dementia, studies combining magnetoencephalography (MEG) and PET have demonstrated a slowing of neurophysiological signalling in response to the cortical accumulation of amyloid-β and phosphorylated tau proteins.^2,3^ Compared to healthy older adults, such slowing is indicated by increased low-frequency activity in the delta (2–4 Hz) and theta (4–8 Hz) bands, alongside decreased signalling in the faster alpha (8–12 Hz) and beta (15–30 Hz) bands, in the brains of individuals with Alzheimer’s disease. Neurophysiological slowing in Alzheimer’s disease is associated with the severity of cognitive impairments,^2^ predicts post-mortem proteinopathy on histopathology^4^ and is shaped by the topography of cortical neurotransmitter systems.^5^

Until recently, much less has been known about how Alzheimer’s disease pathology affects neurophysiology in the pre-clinical stages of the disease, when individuals are asymptomatic and therapeutic interventions more likely to be efficacious. Research in rodent and *in vitro* models has suggested that early exposure to Alzheimer’s disease proteinopathy shifts neurophysiological signalling into a regime of hyperexcitability, which then inverts towards hypoexcitability as amyloid-β and tau continue to accumulate and circuit function is degraded.^6^ Two recent MEG-PET studies of cognitively unimpaired older adults suggest that this non-linear trajectory is captured by a shift from neurophysiological acceleration to slowing in the human brain,^7,8^ with emphasis on signalling changes in the alpha band.

In their study published recently in *Brain Communications*, Scheijbeler *et al*.^9^ add valuable findings to this literature by investigating longitudinal associations between the accumulation of amyloid-β and neurophysiological signalling in cognitively unimpaired participants. At baseline, they found that deposition of amyloid-β was related to higher power in the theta band. Baseline amyloid-β burden in cortical regions sensitive to early Alzheimer’s disease pathology also predicted longitudinal increases in theta power. These associations were generally mirrored by an inverted pattern of effects on theta-frequency connectivity and beta-band power, though these secondary effects were either not subjected to multiple comparisons correction or did not survive statistical significance thresholds. Taken together, these findings suggest that amyloid-β deposition may lead to neurophysiological slowing in the asymptomatic stages of Alzheimer’s disease.

This is in apparent conflict with the previous reports^7,8^ of a non-linear effect of amyloid-β on neurophysiological signalling, but several factors could explain the discrepancy. Most notably, while the key findings in this study involved theta-frequency activity, neither of the previously mentioned studies in this area found amyloid-related alterations in the theta band.^7,8^ Nakamura *et al*.^8^ saw that theta activity did not differ between asymptomatic adults with and without amyloid-β pathology, but was instead related to MRI-derived measures of hippocampal degeneration and general cognitive decline. A similar relationship between theta signalling alterations and general disease status has also been demonstrated in the clinical stages of Alzheimer’s disease.^10^ While Scheijbeler *et al*.^9^ did collect detailed neuropsychological testing data from their participants, they did not test relationships between these cognitive scores and the reported theta band alterations. Additionally, while amyloid-β accumulation is considered a key step in the pathological cascade of Alzheimer’s disease, it is only minimally predictive of future clinical progression. Scheijbeler *et al*.^9^ did not consider factors that are more clinically predictive, such as the presence of tauopathy, as moderating influences on the reported relationships. This could help to determine which associations reported in this study are relevant for conversion to the clinical stages of MCI and dementia and is a clear next step for this area of research. Finally, it is also possible that differences in the data acquisition procedures, such as the use of eyes-open versus eyes-closed protocols, could have differentially affected spectral analyses between these studies.

Potential discrepancies notwithstanding, the study by Scheijbeler *et al*.^9^ provides key new insights into the effects of Alzheimer’s disease proteinopathy on cortical neurophysiology by extending this line of inquiry into the longitudinal accumulation of amyloid-β in asymptomatic participants. Though this area of research is nascent, it has the potential to deliver essential new knowledge on the neuropathophysiological effects of Alzheimer’s disease and help derive new clinical markers for repeatable and non-invasive disease diagnosis, prognosis and tracking.

## Data Availability

No data were generated for this commentary.
